# Exploration on Varying Patterns of Morphological Features and Quality of Armeniacae Semen Amarum in Rancid Process Based on Colorimeter, Electronic Nose, and GC/MS Coupled With Human Panel

**DOI:** 10.3389/fphar.2022.599979

**Published:** 2022-05-03

**Authors:** Yuanyang Shao, Huirong Chen, Hongxin Lin, Huishang Feng, Jianting Gong, Guangzhao Cao, Weifeng Hong, Yuebao Yao, Huiqin Zou, Yonghong Yan

**Affiliations:** ^1^ School of Chinese Materia Medica, Beijing University of Chinese Medicine, Beijing, China; ^2^ Department of Chinese Medicine, The First Affiliated Hospital of Zhengzhou University, Zhengzhou, China; ^3^ Clinical Study Department, Beijing Highthink Pharmaceutical Technology Service Co., Ltd., Beijing, China; ^4^ Department of Dermatology, Dongzhimen Hospital Beijing University of Chinese Medicine, Beijing, China; ^5^ Chinese Medicine Resource Research Center, Beijing Institute of Clinical Pharmacy, Beijing, China

**Keywords:** armeniacae semen amarum, human panel, colorimeter, electronic nose, gas chromatography–mass spectrometry

## Abstract

In recent years, the domestic and international trade volumes of Chinese medicinal materials (CMMs) keep increasing. By the end of 2019, the total amount of exported CMMs reached as high as US $1.137 billion, while imported was US $2.155 billion. A stable and controllable quality system of CMMs apparently becomes the most important issue, which needs multifaceted collaboration from harvesting CMMs at a proper season to storing CMMs at a proper temperature. However, due to imperfect storage conditions, different kinds of deteriorations are prone to occur, for instance, get moldy or rancid, which not only causes a huge waste of CMM resources but also poses a great threat to clinical medication safety and public health. The key issue is to quickly and accurately distinguish deteriorated CMM samples so as to avoid consuming low-quality or even harmful CMMs. However, some attention has been paid to study the changing quality of deteriorated CMMs and a suitable method for identifying them. In this study, as a medicine and food material which easily becomes rancid, armeniacae semen amarum (ASA) was chosen as a research objective, and experimental ASA samples of different rancidness degrees were collected. Then, various kinds of analytical methods and technologies were applied to explore the changing rules of ASA quality and figure out the key indicators for the quality evaluation of ASA in the rancid process, including the human panel, colorimeter, electronic nose, and GC/MS. This study aims to analyze the correlation between the external morphological features and the inner chemical compounds, to find out the specific components from “quantitative change” to “qualitative change” in the process of “getting rancid,” and to discover the dynamic changes in the aforementioned key indicators at different stages of rancidness. The results showed since ASA samples began to get rancid with the extension of storage time, morphological features, namely, surface color and smell, changed significantly, and the degree of rancidness further deepened at the same time. Based on macroscopic identification accomplished *via* the human panel, ASA samples with varying degrees of rancidness were divided into four groups. The result of colorimeter analysis was in agreement with that of the human panel, as well as the determination of the amygdalin content and peroxide value. Moreover, there were obvious differences in the amygdalin content and peroxide value among ASA samples with different rancidness degrees. With a higher degree of rancidness, the content of amygdalin decreased, while the peroxide value increased significantly. The rancidness degree of ASA has a negative correlation with the amygdalin content and a positive correlation with the peroxide value. The newly discovered nonanal and 2-bromopropiophenone in rancid ASA samples may be the key components of “rancidity smell,” and these two components would be the exclusive components that trigger “quantitative change” to “qualitative change” in the process of rancidness of ASA. This study sheds light on studying the internal mechanism of “rancidness” of CMMs and provides an important basis for the effective storage and safe medication of easy-to-get rancid herbs, and it also plays an important foundation for the establishment of a stable and controllable quality system for CMMs.

## Introduction

The production capacity of Chinese medicinal materials (CMMs) in China continues to increase. The survey showed that since 2004, the production capacity of 200 commonly used CMMs had increased from the initial 1.0473 million tons to 3.1751 million tons in 2019 (Jia et al., 2020; Liu et al., 2020). However, the quality of CMMs is always affected by external factors (oxygen, mold, etc.) and internal factors (water content, oils, sugars, etc.) during storage, and there are many physical and chemical changes, which mostly would cause deterioration such as moth, mildew, and rancidness. Rancidness is one of the main deterioration phenomena in the storage of CMMs. It refers to the fact that under the condition of specific natural factors, fat, volatile oil, sugar, and other ingredients contained in the CMMs would slowly spill over on/out of the surface, directly resulting in CMMs to appear oily in the moist state, the texture becomes different obviously, the color becomes darker or accompanied by the phenomenon of “rancidity smell” (Gong et al., 2020a; Shaiqah et al., 2020). Once getting rancid, the active components of CMMs are affected, and there is a certain degree of loss of medicinal properties, which is harmful to clinical safety and efficacy, and even produces toxicity (Olatunji et al., 2018). However, for the changes in medicinal materials and the loss of quality during storage, most of the current research studies are limited to the detection of one or a class of chemical compounds, which cannot represent the overall quality of CMMs, and there are deficiencies (Xu et al., 2017; Beckerman and Persaud, 2019; Wang and Zhu, 2019; Zhang et al., 2019; Yu et al., 2021), this will, to a large extent, cause “deteriorated CMMs” being consumed in the markets and pharmacies, seriously threatening public health (Xin et al., 2015; Dong et al., 2019; Xiong et al., 2019; Zhang, 2020).

The quality change of CMMs during storage can be shown as the change of morphological and sensory features, including shape, density, texture, color, and smell. Along with the physical properties, chemical substances also vary in three aspects, namely, type, content, and ratio (Gong et al., 2020a). However, what is the relationship between them? What is the mechanism of variety in the process of rancidness? Is there a specific index component that can predict the quality of herb medicine from “quantitative change” to “qualitative change?” This kind of research related to the problems mentioned earlier would without doubt be beneficial to the early identification of CMM deterioration, the formulation and prediction of the storage period, and the safe and effective clinical medication. However, there are few reports in related research fields.

Fuzzy mathematics is based on the theory of the membership degree and membership function in fuzzy mathematics. It uses mathematical methods to abstract the constraints of multiple factors in sensory quality so as to establish an ideal evaluation model to reflect the essential characteristics and dynamic processes of the sample (Lu et al., 2021). This method makes up for the defects of traditional sensory evaluation methods which are vague, unable to be quantitatively described, and difficult to distinguish clear boundaries. Fuzzy mathematics can quantify and digitalize sensory indexes and is an important tool in the study of sensory quality. At present, this method has been mainly applied in many fields but rarely in the field of CMM quality evaluation (Ali et al., 2016; Putti et al., 2017; Wang et al., 2019).

The color of herbal medicine has always been a key indicator of traditional macroscopic identification, and it plays an important role in identifying the authenticity of CMMs. To a certain extent, the difference in color can directly reflect the quality of CMMs (Qi et al., 2016). The application of colorimetry enables the quantification and standardization of the “color” in the identification of CMMs. Based on the colorimetric theory, the digital methods of color indexes including cross section, profile, and powder of CMMs can be established, which provides a new idea and method for the objectification and digitization of sensory color in CMM identification. Scholars have studied different herb medicines using color digitization methods and found that CMM color has a certain correlation with the intrinsic quality (Yin et al., 2013; YongXia et al., 2019).

Odor is one of the important traits of CMMs. The smell of CMMs is closely related to the chemical components contained in it, and it can directly reflect the inherent quality of the medicine. Electronic nose is an intelligent sensory instrument that simulates the olfactory cells of humans and animals with a sensor array to analyze, identify, and detect complex odors and volatile components (Shi et al., 2018). This technology overcomes the shortcomings of strong subjectivity and low accuracy of traditional sensory evaluation in terms of nose smell and can quickly, sensitively, accurately, and comprehensively reflect the overall odor characteristics of CMMs. It has advantages in odor recognition and is now widely used in odor analysis and quality evaluation of CMMs (Zou et al., 2015; Zhou et al., 2017; Xu et al., 2018).

The headspace solid-phase microextraction method (SPME) integrates sampling, extraction, and concentration techniques and has the advantages of short processing time, no organic solvents, and true reflection of the volatile components of the samples (Burzynski-Chang et al., 2018). Gas chromatography–mass spectrometry (GC/MS) is widely used in the identification of volatile components (Beale et al., 2018). SPME–GC/MS have outstanding advantages in the identification and analysis of plant volatile components (Mentana et al., 2019; Nespor et al., 2019; Rivers et al., 2019). The volatile components of armeniacae semen amarum (ASA) are changed after getting rancid, followed by releasing “rancidity smell.” In this study, SPME–GC/MS was employed to track the chemical components in the smell of ASA to find the internal composition of the “rancidity smell.”

ASA samples are rich in fats and are prone to get rancid. In addition, the active ingredient amygdalin will be decomposed under certain conditions to produce the toxic component hydrocyanic acid, and excessive use may cause poisoning or even death (Shragg et al., 1982; Gong et al., 2020b). Therefore, as a bulk medicine and food material which is prone to get rancid during storage, ASA was selected as the representative objective, and its rancidity characteristics were studied by human panel methods, color digitization methods, electronic nose fingerprinting, and SPME–GC/MS technology. Herein, the goals of this study are 1) to find out the relationship between the characteristics and internal chemical components in the process of rancidness, 2) to discover the regulation of rancidness under specific storage conditions, and 3) to figure out specific indicators that can predict the quality of medicinal materials from “quantitative change” to “qualitative change.” The results of this study have important reference implication for the effective storage and clinical medication, the analysis of the composition changes in the process of rancidness, and the interpretation of the internal mechanism of ASA and other easy-to-be-deteriorated herb medicine. This study provides ideas and methods for the establishment of a stable and controllable quality system for CMMs.

## Materials and Methods

### Plant Material

Three kinds of ASA samples, namely, raw, peeled, and fried, were collected from Inner Mongolia (116.62°N, 40.21°E) and Hebei (117.20°N, 40.51°E) (Table 1), and they were identified as *Prunus armeniaca* L. var. *ansu* Maxim. and *Prunus mandshurica* (Maxim.) Koehne by Prof. Yonghong Yan (Beijing University of Chinese Medicine, Beijing, China). The samples were evenly mixed and stored in two different environments: one was stored in Beijing, China [116.32°E, 39.89°N, 25 ± 2°C, 60 ± 10% relative humidity (RH)] (Zheng and Liu, 2008) (sampled every 3 months), and the other in a drug stability test chamber (40°C, 75% RH) in a high-temperature and -humidity environment (sampled every 10 days). The sampling frequency was determined according to the preliminary experiment of the research group (Gong, 2017; Zhao et al., 2017).

**TABLE 1:** List of sample information.

Storage condition	Storage time	Sample number		
		Raw	Peeled	Fried
Room temperature, ventilation room of the Beijing laboratory	0 month	S1	D1	C1
	3 months	S2	D2	C2
	6 months	S3	D3	C3
	9 months	S4	D4	C4
	12 months	S5	D5	C5
	24 months	S6	D6	C6
	27 months	S7	D7	C7
	30 months	S8	D8	C8
	33 months	S9	D9	C9
	36 months	S10	D10	C10
Constant temperature and humidity chamber (40°C, 75% RH)	10 days	S11	D11	C11
	20 days	S12	D12	C12
	30 days	S13	D13	C13
	40 days	S14	D14	C14
	50 days	S15	D15	C15
	60 days	S16	D16	C16
	70 days	S17	D17	C17
	80 days	S18	D18	C18
	90 days	S19	D19	C19
	100 days	S20	D20	C20
	110 days	S21	D21	C21
	120 days	S22	D22	C22

### Instruments and Reagents

The instruments and reagents used were listed as follows: a Bruker 320 GC-MS system (Bruker corporation, Nasdaq, United States), DB-5ms Intuvo columns (0.25 µm × 0.25 mm × 30 m, Agilent Corp, California, United States), a CM-5 Spectrophotometer (Konica Minolta Holdings Co., Ltd., Tokyo, Japan), a Heracles NEO α-FOX3000 electronic nose (Alpha M.O.S. Corp, Toulouse, France), a Waters Alliance HPLC system (Waters Corp, Massachusetts, United States), an Agilent ZORBAX SB-C18 column (4.6 mm × 250 mm × 5 µm particle size, Agilent Corp, California, United States), and an amygdalin reference substance (Nature Standard corporation, ST02010120MG, Shanghai, China).

## Methods

### Sensory Evaluation of ASA

A fuzzy mathematics method was used to establish sensory evaluation standards for the surface color, section color, odor, and degree of oil moistening of the ASAs. Different evaluation criteria were divided into four grades, and the binary comparison determination method was used to determine the proportion of different standards in the comprehensive evaluation (Lu et al., 2021). A total of 10 previously trained professionals were invited to comprehensively evaluate the rancidness degree of ASA according to this standard, and the results were summarized and analyzed (Schlossareck and Ross, 2019).

### Color Determination of ASA

The method of color digitization was applied to evaluate the ASAs with different degrees of rancidness. Powder: the sample was crushed, passed through a No. 2 sieve, and mixed evenly; an an appropriate amount of the powder was added to the color measuring dish, covered with a glass slide, and sealed. Detection conditions: starting and ending wavelength range: 380–780 nm, scanning speed: 600 nm/min, slit width: 1 nm, illumination source: D65, and field of view: 10°. Different machine learning algorithms were used to distinguish the degree of rancidness of ASA according to the powder color of the sample, and two verification methods of a ten-fold crossover and an external test set were used to verify (Zheng and Liu, 2008). Section: ASA was divided into two halves along the seed ridge, and the color of the ASA section was determined by using the CM-5 spectrophotometer. Measurement conditions: starting and ending wavelength range: 360–740 nm, illumination source: D65, field of view: 10°, illumination system: specular component included reflection, and test area: 3 mm. The analysis method was the same as the powder color analysis method.

### Determination of ASA Odor

The sample was crushed, passed through a No. 2 sieve, accurately weighed 0.30 g, put into the headspace sample bottle, and sealed with a gland. Measurement conditions: incubation time: 600 s, incubation temperature: 45°C, oscillation speed: 250 r·s-1, injection volume: 2,500 μL, carrier gas: compressed air, flow rate: 150 ml·min-1, data acquisition time: 120 s, acquisition cycle: 1 s, and purge time: 600 s.

### Determination of Amygdalin Content in ASA

Preparation of sample solution: the power was sifted through the No. 2 sieve, weighed 0.25 g precisely, put in a conical bottle, and 25 ml methanol was added into the bottle, weighed, sonicated 30 min, weighed again, and the missing mass was supplied with methanol, shaken well, filtered, and 5 ml filtrate was transferred to a 25-ml volumetric bottle, and 50% methanol was added to dilute to the scale line, and shaken well. Preparation of reference solution: amygdalin was diluted with methanol at 203.20 μg/ml, mobile phase: methanol–water (20:80), flow rate: 1 ml min-1, detection wavelength: 207 nm, and injection volume: 10 μl.

### Determination of Peroxide Value in ASA

The peroxide value determination method was based on the method of the 2015 edition of “Chinese Pharmacopoeia” (Zhang et al., 2019).

### Identification of Volatile Components in ASA

The sample was crushed, sifted through No. 2 sieve, accurately weighed 2.00 g, put into a 20-ml headspace sample bottle, sealed, extracted in a water bath at 60°C for 40 min, and activated for 20 min at 270°C. The heating program: 50°C for 5 min, heating up to 290°C at the rate of 10°C/min for 11 min, injection temperature: 270°C, EI ion source temperature: 200°C, transmission line temperature: 250°C, and mass spectrometry scanning range: 45–800 Da. Check the NIST147 database and refer to the related reference (Feng et al., 2018; Kalagatur et al., 2018; Sai Latha et al., 2018; Machado et al., 2019; Ahmad et al., 2020) to identify the volatile components.

## Results and Discussion

### Sensory Evaluation of ASA

**FIGURE 1:**
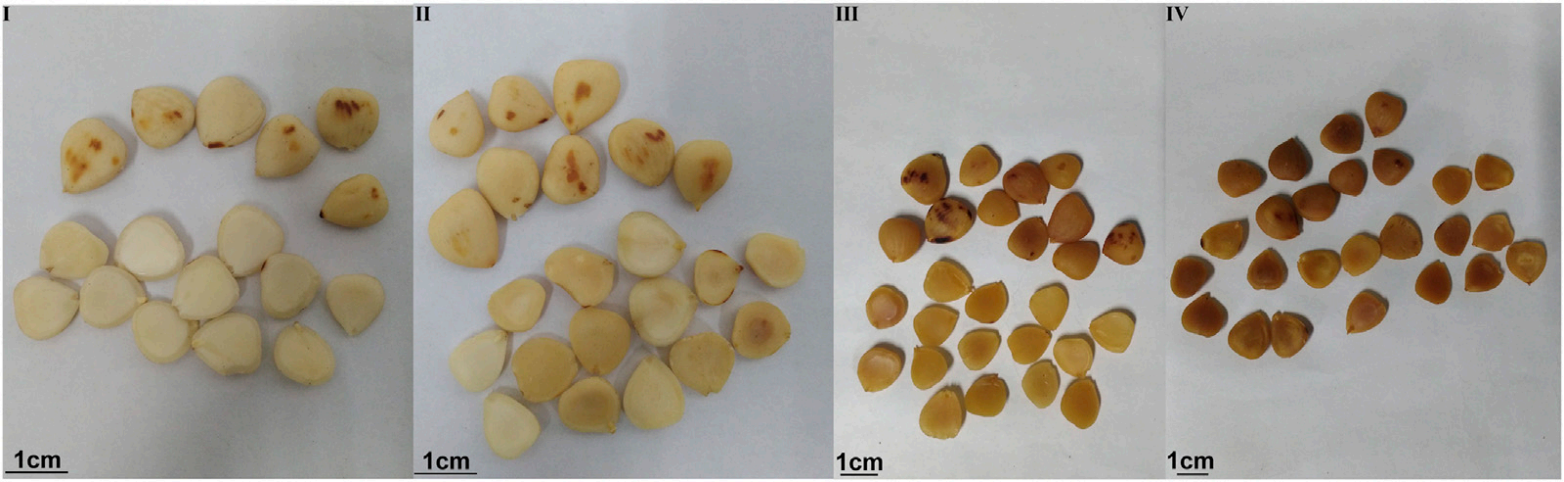
Samples of different rancidness levels (the temperature and relative humidity of grade Ⅰ samples: room temperature and 46.33%, the storage time of grade Ⅰ samples: 1 y; the temperature and relative humidity of grade Ⅱ samples: 40°C and 75%, the storage time of grade Ⅱ samples: 10–50 days; the temperature and relative humidity of grade Ⅲ samples: 40°C and 75%, the storage time of grade Ⅲ samples: 60–70 days; and the temperature and relative humidity of grade Ⅳ samples: 40°C and 75%, the storage time of grade Ⅳ samples: 80–110 days).

**FIGURE 2:**
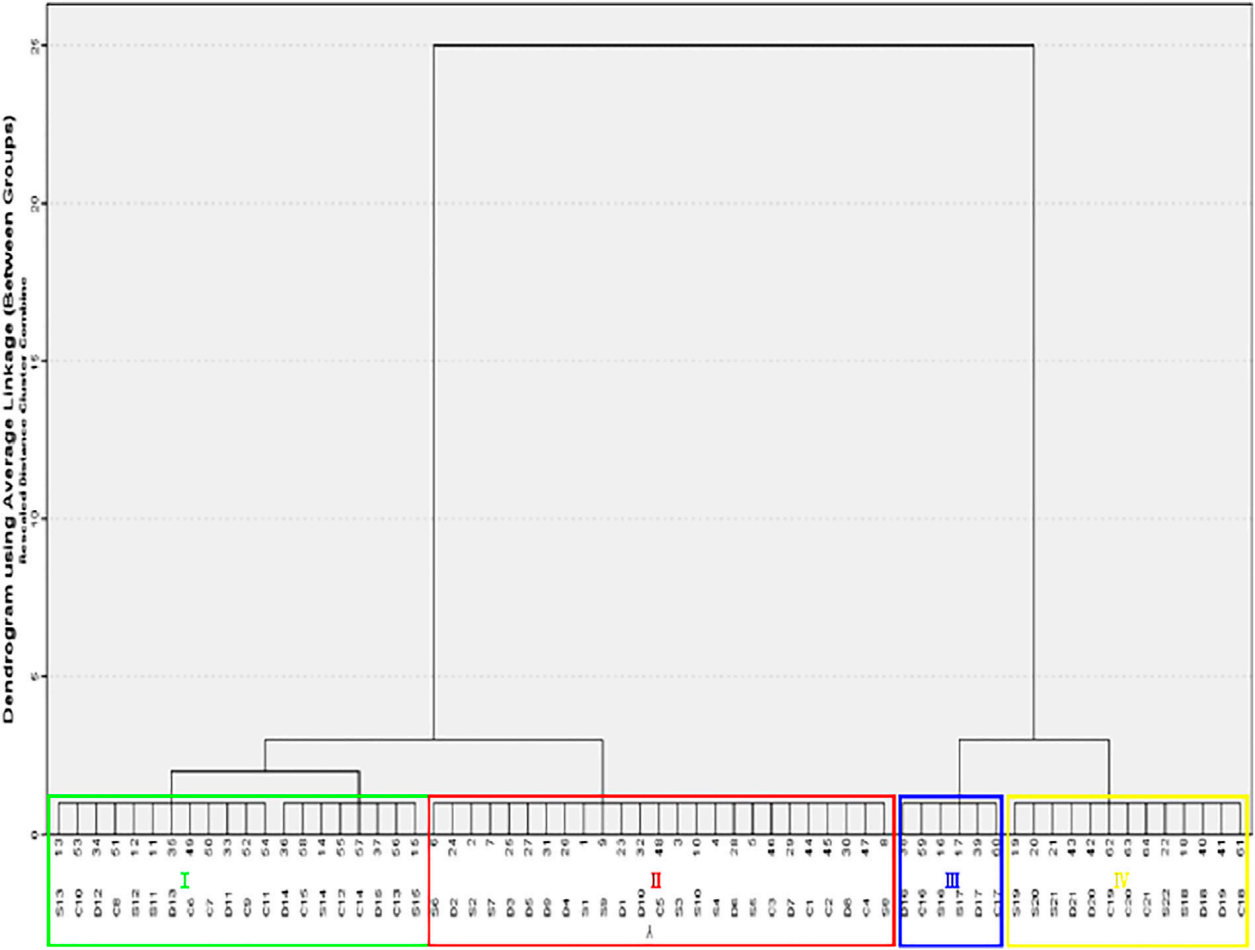
Cluster map of different rancidness sample.

The three processed products of ASA samples were evaluated according to surface color, section color, smell, and oily state by an evaluation team composed of 10 evaluators. The results showed that according to the fuzzy sensory evaluation method and evaluation standard, the rancidness degree of ASA can be divided into four grades: Ⅰ–Ⅳ (Figures 1, 2). The results found that there were significant differences in the macroscopic characteristics of ASA with different degrees of rancidness, which could be divided into four grades according to their morphological characteristics.

### Determination of ASA Odor

#### Powder

**FIGURE 3:**
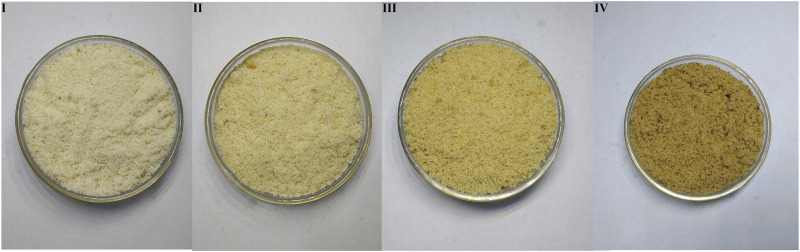
Powder color of samples with different rancidness levels (the temperature and relative humidity of grade Ⅰ samples: room temperature and 46.33%, the storage time of grade Ⅰ samples: 1 y; the temperature and relative humidity of grade Ⅱ samples: 40°C and 75%, the storage time of grade Ⅱ samples: 10–50 days; the temperature and relative humidity of grade Ⅲ samples: 40°C and 75%, the storage time of grade Ⅲ samples: 60–70 days; and the temperature and relative humidity of grade Ⅳ samples: 40°C and 75%, the storage time of grade Ⅳ samples: 80–110 days).

The human panel method has the shortcomings of strong subjectivity and low accuracy. In this experiment, the color digitization method was used to determine the powder color of ASA, which was made up for the low accuracy of color feature evaluation in the human panel method, and the rancidness grade of ASA verified preliminarily divided by the human panel method. It was found that among the 12 classification methods, the model established by the Naive Bayes algorithm, and the positive judgment rate of the two verification methods was above 85% (Zheng and Liu, 2008). The results showed that the model established by using the Naive Bayes algorithm could be used to determine the degree of rancidness of ASA. Further analysis found that the results were consistent with the human panel results (Figure 3). The color determination method of ASA powder established in this study combined with the model established by the Naive Bayes algorithm could be applied to determine the varying rancid degrees of ASA.

#### Profile

**FIGURE 4:**
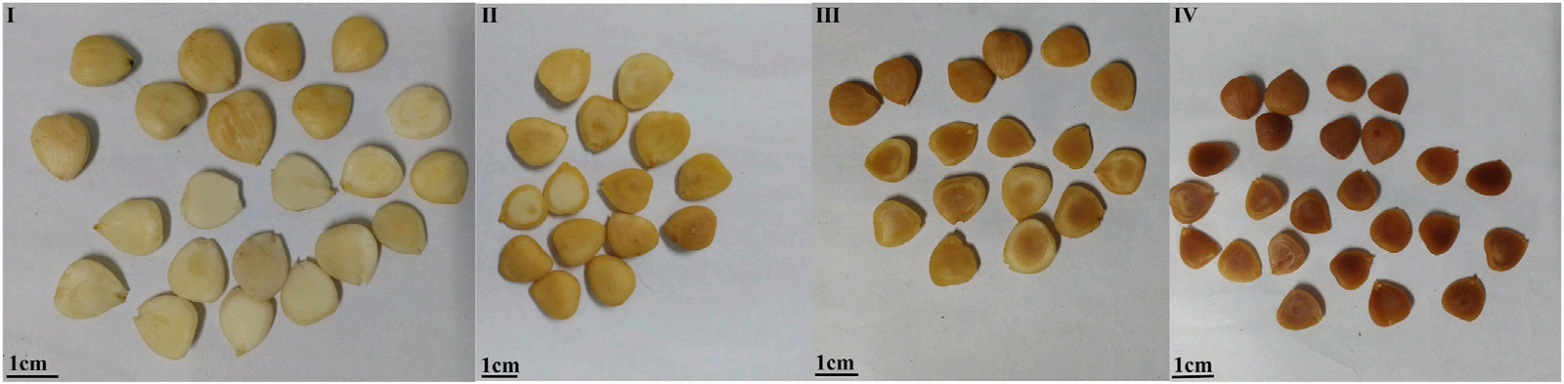
Profile color of samples with different degrees of rancidness (the temperature and relative humidity of grade Ⅰ samples: room temperature and 46.33%, the storage time of grade Ⅰ samples: 1 y; the temperature and relative humidity of grade Ⅱ samples: 40°C and 75%, the storage time of grade Ⅱ samples: 10–50 days; the temperature and relative humidity of grade Ⅲ samples: 40°C and 75%, the storage time of grade Ⅲ samples: 60–70 days; and the temperature and relative humidity of grade Ⅳ samples: 40°C and 75%, the storage time of grade Ⅳ samples: 80–110 days).

In order to further verify the accuracy of the color feature evaluation results in the human panel, this experiment was based on the color digitization method to determine the profile color of ASA, and 12 classification methods were used to distinguish the degree of rancidness of ASA. The results presented that the correct judgment rate of logistic and multiple layer perceptron algorithms was higher. The results demonstrated that both logistic and multiple layer perceptron algorithms could be applied to determine the rancid degree of ASA. In addition, the results were consistent with the results of the human panel (Figure 4). The color measurement method of the ASA section established in this study, combined with logistic and multiple layer perceptron algorithms, can be used to distinguish the rancidness degree of ASA.

### Odor Fingerprint of ASA

**FIGURE 5:**
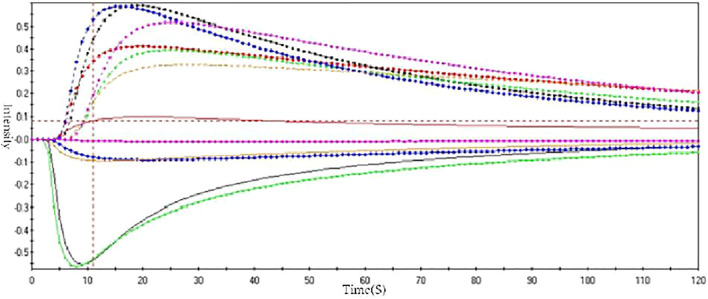
Response of electronic nose sensor array to ASA odor.

**FIGURE 6:**
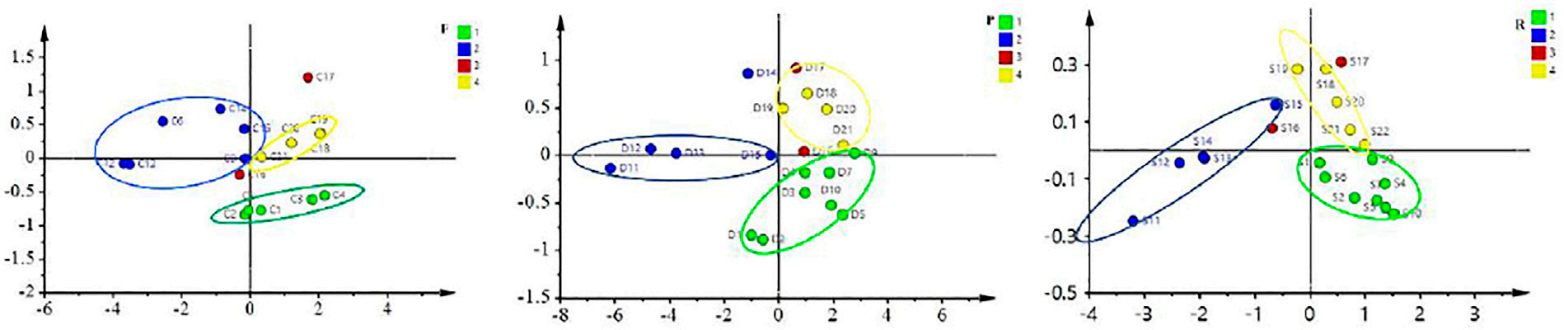
PLS-DA analysis chart of different rancidness of ASA (1–4: Ⅰ–Ⅳ).

To verify the accuracy of odor evaluation results in the human panel, the electronic nose was used to analyze the smell of ASA with different degrees of rancidness. Through the optimization of sensors and algorithms, four sensors and the logistic algorithm were finally selected (Figure 5). The data were sorted out and analyzed by PCA. The results showed that ASAs with different degrees of rancidness were roughly distributed in different areas. The results of odor analysis were consistent with the human panel results and also showed no difference with the results of color digital measurement. The results verified the accuracy of the human panel and color digital measurement results. The results evidenced that this method could be able to determine and identify the odor of ASA with different degrees of rancidness (Figure 6). Furthermore, the results of pharmacological studies showed that there were significant differences in the cough-relieving effects of ASAs in varying degrees of rancidity. Compared with the blank control group, the peeled and fried products without and slightly getting rancid had a significant cough-relieving effect, but the completely rancid-peeled products had no cough-relieving effect. Furthermore, combined with the data of the electronic nose, the heavier the sensory smell of ASA, the less obvious the cough relief effect (Cao et al., 2020).

### Determination of Amygdalin Content in ASA

**FIGURE 7:**
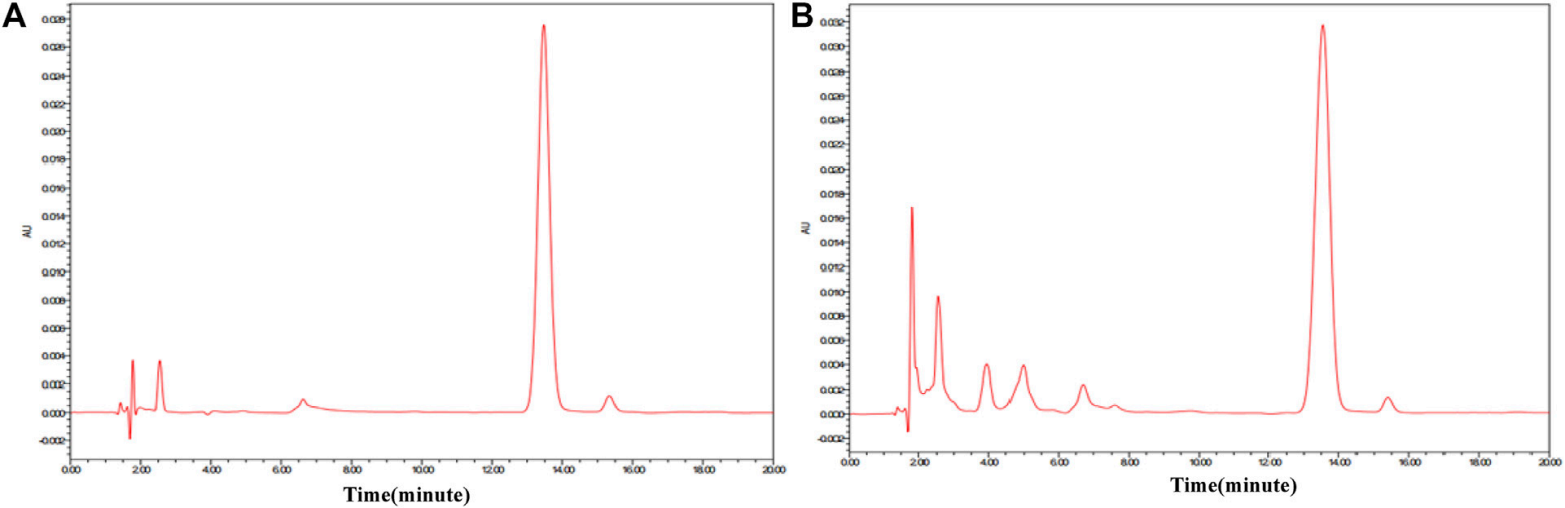
HPLC of amygdalin reference substance and ASAs. **(A)** Amygdalin reference substance. **(B)** ASA.

In order to find out the relevance between the macroscopic characteristics and chemical components of ASAs during the rancidness process, the effective component amygdalin was determined by HPLC. The results displayed that there were differences in the content of amygdalin in ASA with different degrees of rancidness (Figure 7). The rancidness degree of the samples was divided according to the content of amygdalin, and the result was in accordance with the results of the human panel, indicating that there was a certain correlation between the characteristics and chemical components in the process of rancidness of ASA. In addition, we further analyzed and found that the content of amygdalin with grade I of the raw samples conformed to the standard of the Chinese Pharmacopoeia (above 3.0%), and the content of amygdalin in partial products with grade Ⅱ reached the standard of the Chinese Pharmacopoeia. The content of amygdalin with grades Ⅰ and Ⅱ of some peeled samples was not up to the standard of Chinese Pharmacopoeia (above 2.4%). In the samples with the degree of rancidness in the fired products, the content of amygdalin in some samples reached the Chinese Pharmacopoeia standard (above 2.4%). Notably, the contents of amygdalin in the samples of the grade Ⅲ and grade Ⅳ rancidness of the three processed products did not meet the Chinese Pharmacopoeia standards. The analysis results indicated that the amygdalin content of some ASA with the rancidness degree of grades Ⅰ and Ⅱ reached the Chinese Pharmacopoeia standard, and there was no significant difference, and they could continue to be used for medicinal purposes. The samples of the grade Ⅲ and grade Ⅳ rancidness of the three processed products, whose content of amygdalin decreased significantly, and it did not meet the Chinese Pharmacopoeia standards and could not continue to be used as medicine. In summary, according to the grade of rancidness established in this study, the amygdalin content of samples with rancidness grade Ⅱ was lower than that of samples of grade Ⅰ as a whole, and there was no significant difference between grade Ⅰ and grade Ⅱ samples; some samples can continue to be used for medicinal purposes. The content of amygdalin in the samples of grades Ⅲ and Ⅳ cannot reach the standard of Chinese Pharmacopoeia and cannot be used as medicine. The results were consistent with the results of the human panel, indicating that there was a certain correlation between the macroscopic characteristics and chemical components of ASA in the process of rancidness.

### Determination of Peroxide Value in ASA

**FIGURE 8:**
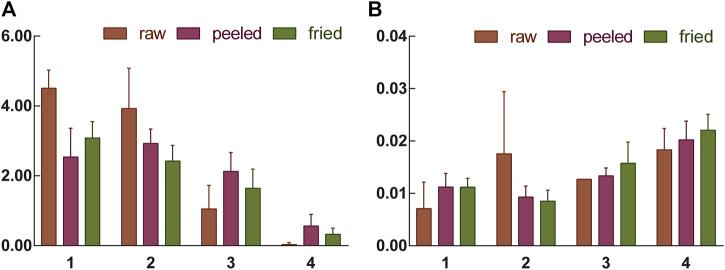
Amygdalin content **(A)** and peroxide value **(B)** of samples with different rancidness levels (1–4: Ⅰ–Ⅳ).

In order to further explore the correlation between macroscopic characteristics and chemical characteristics of ASAs during rancidness, the peroxide value of samples was determined. The results showed that the peroxide value of the ASAs with rancidness is significantly different, and the peroxide value of the samples gradually increased as the degree increased (Figure 8). According to the level of peroxide value, the rancidness grade of ASAs was classified, and the results were consistent with the results of the human panel. Combined with the classification of the degree of rancidness in the human panel, further analysis found that the peroxide values of the samples with human panel grades Ⅰ and Ⅱ were lower than 0.012, and the peroxide values of samples Ⅲ and Ⅳ were both higher than 0.012, which was significantly higher than the samples whose rancidness level was Ⅰ and Ⅱ. This result proved that the peroxide values of ASAs with different rancid grades were significantly different. Moreover, the results suggested that there was a correlation between the macroscopic characteristics and chemical characteristics of ASAs during the rancidness process.

### Identification of Volatile Components in ASA

**FIGURE 9:**
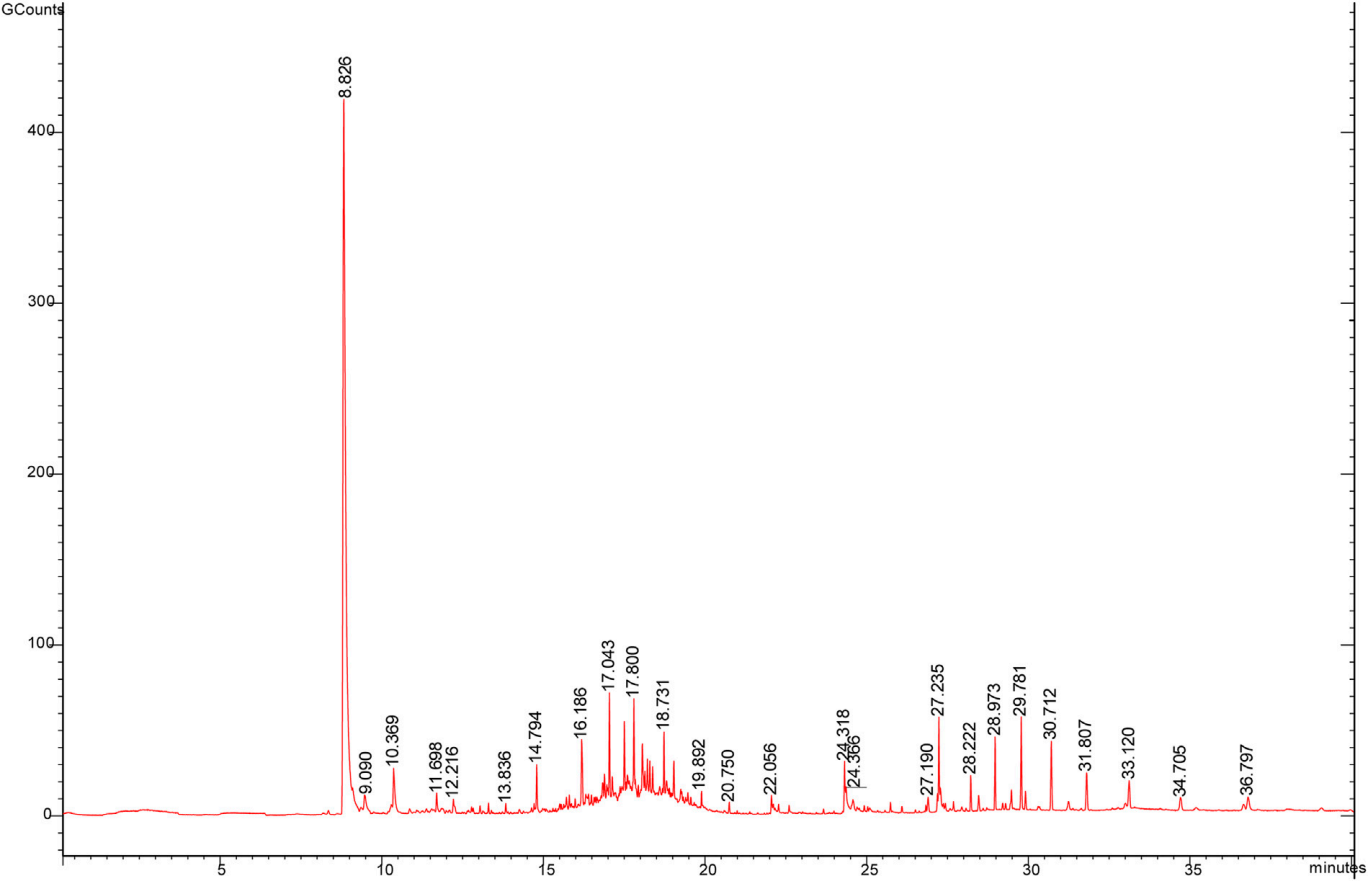
SPME–GC/MS total ion current of ASA.

**FIGURE 10:**
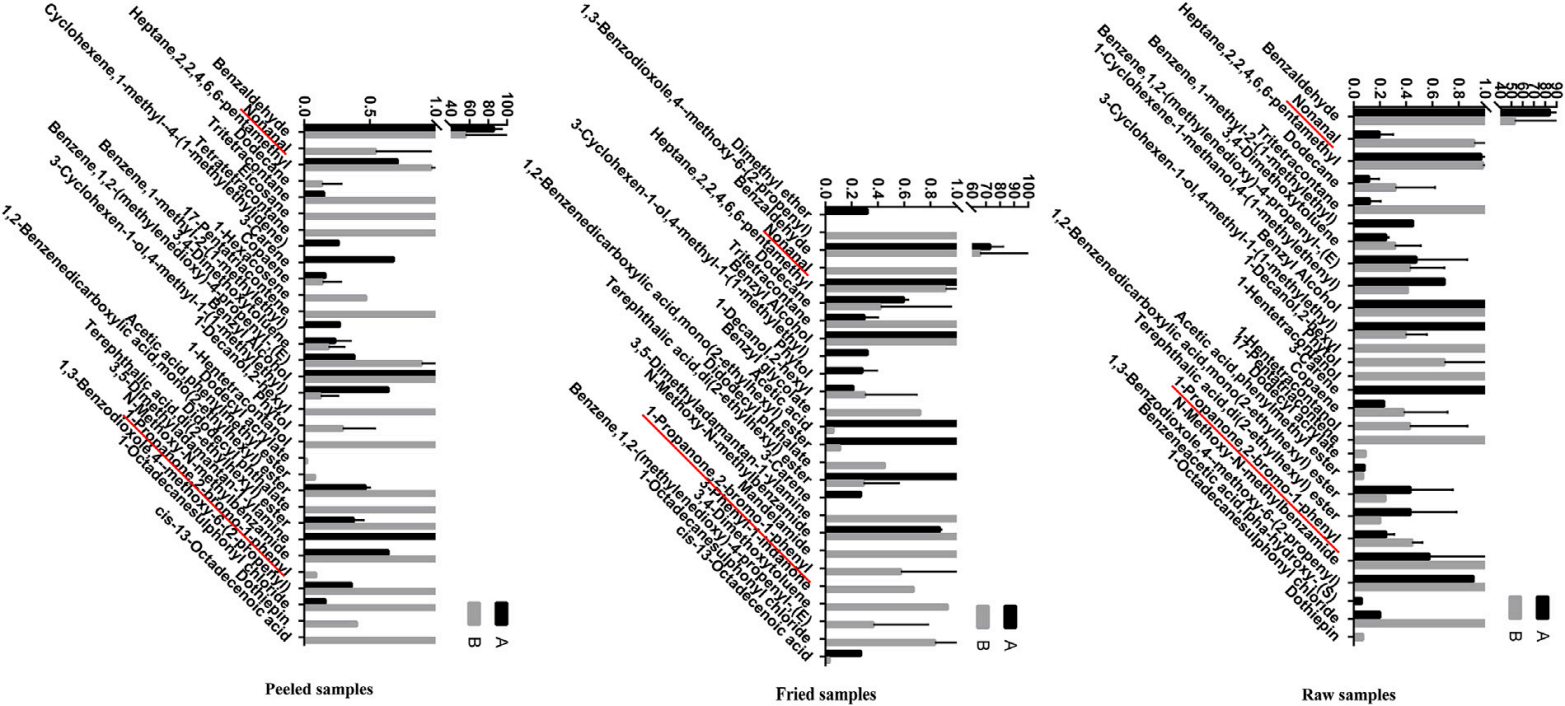
Volatile components in odor and their relative percentages of ASAs (%) (A: no rancidity smell of samples, B: rancidity smell of samples; underline: two components of rancidity smell).

The volatile components of ASAs were alternated after rancidness, releasing “rancidity smell” (Gong et al., 2020b). In order to explore the internal components of “rancidity” in the process of rancidness of ASA, the volatile components were identified by SPME–GC/MS (Figure 9). The results found that 39 compounds were identified in this study (see attachment). It is worth noting that a comparative analysis of the detection results of samples that did not emit “rancidity odor” revealed that two new ingredients were added, namely, nonanal and 2-bromopropiophenone (Figure 10). This result illustrated that the volatile components of ASAs have undergone significant changes during the process of rancidness, and new compounds have been formed.

**FIGURE 11:**
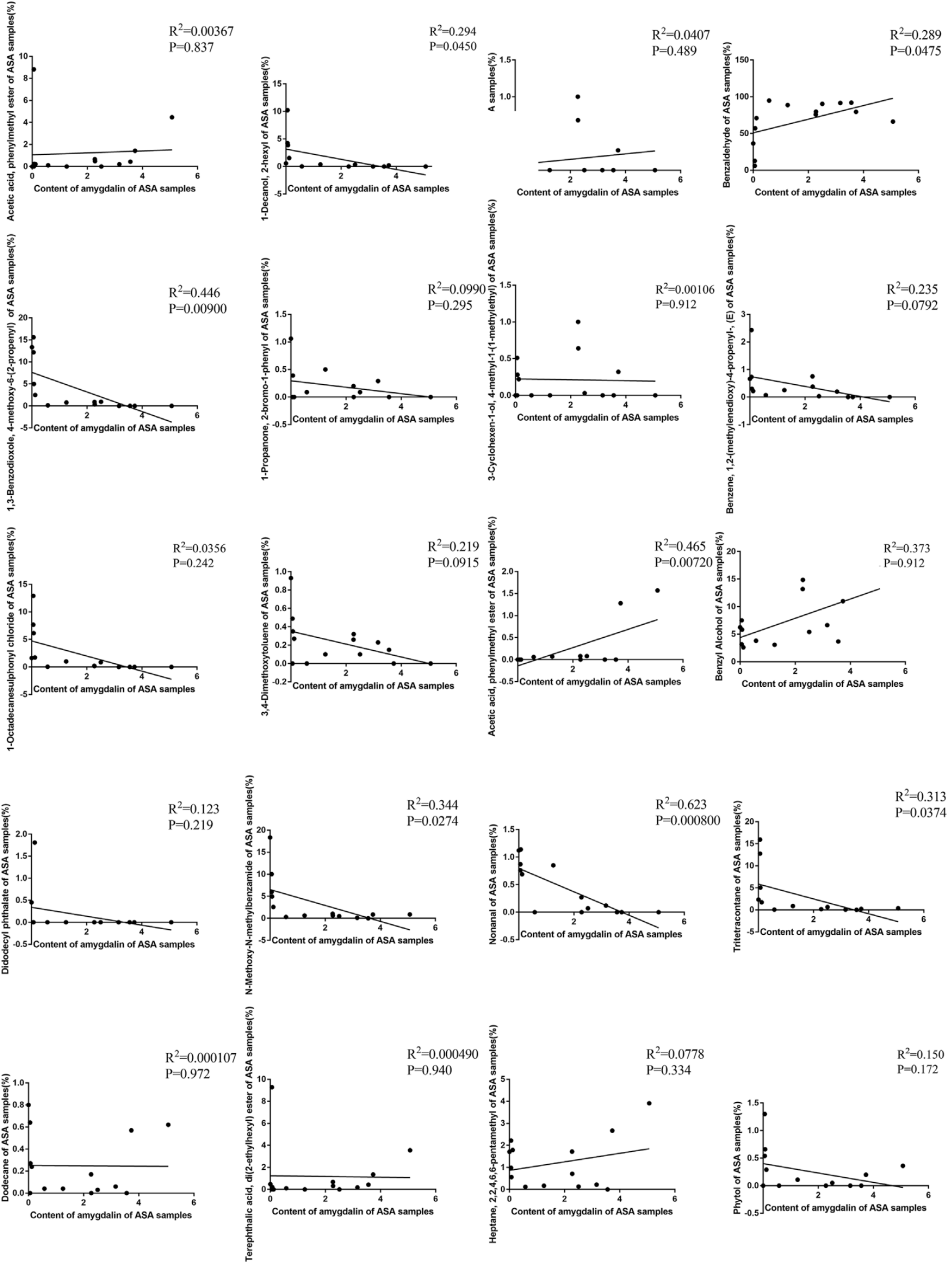
Correlation analysis of ASA composition and volatile components in odor.

To explore the internal relationship between the chemical composition and volatile oils of ASAs, this study conducted a correlation analysis on the detection results about the two sections. The results showed that there was no obvious significant correlation between the relative content of 1,2-benzenedicarboxylic acid, mono(2-ethylhexyl) ester, 1-propanone, 2-bromo-1-phenyl, 3,4-dimethoxytoluene, 3-carene, 3-cyclohexen-1-ol, 4-methyl-1-(1-methylethyl), benzene, 1,2-(methylenedioxy)-4-propenyl-(E), didodecyl phthalate, dodecane, heptane, 2,2,4,6,6-pentamethyl, phytol, terephthalic acid, and di(2-ethylhexyl) ester, and the content of amygdalin. There was an obvious negative correlation between the relative content of 1-decanol, 2-hexyl, 1-octadecanesulfonyl chloride, n-methoxy-n-methylbenzamide, and tritetracontane, and the content of amygdalin. The positive correlation about the relative content of benzaldehyde and benzyl alcohol, and the content of amygdalin was high. There was a very significant negative correlation between the relative content of 1,3-benzodioxole and 4-methoxy-6-(2-propenyl), and the content of amygdalin. The relative content of acetic acid and phenylmethyl ester had a quite significant positive correlation with the content of amygdalin (Figure 11). The results indicated that there was a certain intrinsic relationship between amygdalin and volatile components in the odor of ASA.

## Conclusion

There are abundant species and great demand of medicinal plants in the world, and a large number of CMMs are circulated to the markets and hospitals every year. Due to the imperfect storage conditions, a large quantity of CMMs often deteriorate, such as mildew and rancidness, which not only results in wastage of resources but also poses a serious threat to clinical safety and human health. In this study, to classify the degree of rancidness, we need to objectify the color and smell and find the relationship between the character characteristics and chemical characteristics of ASA with different degrees of rancidness, to discover the rancidness regulation of ASA under specific storage conditions, to analyze the “rancidity smell” components of samples, and to identify specific indicators that predict medicinal materials from “quantity change” to “quality change.” ASA, an easy-to-rancidness medicinal material, was used as a representative, and the human panel method, color digitization method, HPLC, electronic nose fingerprint, and SPME–GC/MS technology were comprehensively used. The results found that the samples could be divided into four grades according to the degree of oil running, and there were significant differences in characters and chemical characteristics among different grades. There was a certain degree of correlation between the character characteristics and chemical characteristics of ASAs in the process of oil running. The peroxide value of grades Ⅰ and Ⅱ was significantly lower than that of grades Ⅲ and Ⅳ samples. In terms of chemical components, the content of amygdalin in some samples of grades Ⅰ and Ⅱ was lower than the standard of Chinese Pharmacopoeia, which could not be used as medicine. The samples whose oil degree is Ⅲ and Ⅳ in terms of characteristics had an amygdalin content lower than the Chinese Pharmacopoeia standard and could not be used for medicine.

Under the conditions of high temperature and humidity, with the extension of storage time, the rancidness of ASA became worse. Its volatile ingredients also began to change. Benzaldehyde was decomposed from amygdalin, and it was one of the main volatile components of ASA without rancidness. After the sample was oiled, the content of aldehydes decreased, the content of benzaldehyde was greatly reduced, and new aldehydes such as nonanal were produced, and in the meantime, the content of ketones, mainly 2-bromopropiophenone, increased. Studies have shown that aldehydes and ketones were closely related to the odor of fat oxidation rancidity, where the end products of fat oxidation and their flavor thresholds were lower than hydrocarbons, furans, and alcohols (Chen et al., 2019). It has also been reported that nonanal is one of the main volatile components that change the smell of soybean oil after oxidation (Nieva-Echevarría et al., 2018). In conclusion, the newly added nonanal and 2-bromopropiophenone may be the main components of the “rancidity smell” after the rancidness of ASA, and it is speculated that these two components could be the predictive indicators of “quantitative change” and “qualitative change” in the process of rancidness of ASA. This study provides important guidelines for the effective storage of CMMs such as ASA and other easy-to-rancidness ones and lays a preliminary foundation for the interpretation of the composition changes and internal mechanisms in the process of rancidness. Moreover, this research has great reference value for the establishment of a stable and controllable quality system of CMMs.

Deteriorating Chinese medicines poses a huge threat to human health. In the 2020 edition of Chinese Pharmacopoeia, a wider range of testing has been formulated, adding aflatoxin, zearalenone, and other toxic components in rancid medicinal materials, as well as peroxide value, rancidity, and other indicators related to the deterioration of CMMs (Commission of Chinese Pharmacopoeia, 2020). Subsequent research needs to combine metabolomics, molecular biology, pharmacology, and toxicology techniques to specifically analyze the internal mechanism of the rancidness process of ASAs and explore the pharmacological effects and changes in toxic components.

## Data Availability

The original contributions presented in the study are included in the article/Supplementary Material; further inquiries can be directed to the corresponding authors.
