# Supplementary material for: Exploration on Varying Patterns of Morphological Features and Quality of Armeniacae Semen Amarum in Rancid Process Based on Colorimeter, Electronic Nose, and GC/MS Coupled With Human Panel
Source: Front Pharmacol. 2022 May 3;13:599979. doi: 10.3389/fphar.2022.599979 (PMC9110824; doi:10.3389/fphar.2022.599979)
Supplement: Supplementary file 1 [file DataSheet1.docx]

**Part One: The** **detail information of the study material**

*Prunus armeniaca* L. var. *ansu* Maxim.

**Accepted name:** Prunus armeniaca L. [Rosaceae]

The link is as follows:

<https://mpns.science.kew.org/mpns-portal/plantDetail?plantId=901800&query=Prunus+armeniaca+L.+var.+ansu+Maxim.&filter=&fuzzy=false&nameType=all&dbs=wcsCmp>

**Taxonomic source and confidence:**World Checklist of Selected Plant Families (WCSP) – Unpublished

The link is as follows:

<https://mpns.science.kew.org/mpns-portal/reference?reference=World%20Checklist:%20unpublished%20records&query=Prunus+armeniaca+L.+var.+ansu+Maxim.&filter=&fuzzy=false&nameType=all>

**Information about this plant:**

Family: [*Rosaceae* Juss.](http://plantsoftheworldonline.org/taxon/urn:lsid:ipni.org:names:30000200-2)

Genus: [*Prunus* L.](http://plantsoftheworldonline.org/taxon/urn:lsid:ipni.org:names:30003057-2)

**Prunus *armeniaca* L.**

This species is accepted, and its native range is Central Asia to N. & Central China.

The link is as follows:

<http://plantsoftheworldonline.org/taxon/urn:lsid:ipni.org:names:729463-1>

***Prunus mandshurica* (Maxim.) Koehne**

**Accepted name:** Prunus mandshurica (Maxim.) Koehne [Rosaceae]

The link is as follows:

<https://mpns.science.kew.org/mpns-portal/plantDetail?plantId=901817&query=Prunus+mandshurica+%28Maxim.%29+Koehne&filter=&fuzzy=false&nameType=all&dbs=wcsCmp>

**Taxonomic source and confidence:**World Checklist of Selected Plant Families (WCSP) - Unpublished

The link is as follows:

<https://mpns.science.kew.org/mpns-portal/reference?reference=World%20Checklist:%20unpublished%20records&query=Prunus+mandshurica+%28Maxim.%29+Koehne&filter=&fuzzy=false&nameType=all>

**Information about this plant:**

Family: [*Rosaceae* Juss.](http://plantsoftheworldonline.org/taxon/urn:lsid:ipni.org:names:30000200-2)

Genus: [*Prunus* L.](http://plantsoftheworldonline.org/taxon/urn:lsid:ipni.org:names:30003057-2)

***Prunus mandshurica* (Maxim.) Koehne**

This species is accepted, and its native range is S. Russian Far East to Korea.

The link is as follows:

<http://plantsoftheworldonline.org/taxon/urn:lsid:ipni.org:names:729939-1>

**Part Two: Sensory Evaluation of ASA Samples**

**Evaluation standard**

Tab. 1 Sensory evaluation standard of raw ASA samples

| Sensory Index | Evaluation Standard | | | |
| --- | --- | --- | --- | --- |
|  | Ⅰ | Ⅱ | Ⅲ | Ⅳ |
| Surface color | yellowish brown (light) | yellowish brown (dark) | dark yellow brown | dark brown |
| Section color | milky | light yellow | brownish yellow | brown |
| Odor | specific aroma | specific aroma (slight) | rancidity (slight) | rancidity |
| Oily condition | none | mild | moderate | severe |

Tab. 2 Sensory evaluation standard of peeled ASA samples

| Sensory Index | Evaluation Standard | | | |
| --- | --- | --- | --- | --- |
|  | Ⅰ | Ⅱ | Ⅲ | Ⅳ |
| Surface color | light yellow | yellow | brownish yellow | yellowish brown |
| Section color | milky | yellow white | brownish yellow | yellowish brown |
| Odor | aroma | aroma (slight) | rancidity (slight) | rancidity |
| Oily condition | none | mild | moderate | severe |

Tab. 3 Sensory evaluation standard of fried ASA samples

| Sensory Index | Evaluation Standard | | | |
| --- | --- | --- | --- | --- |
|  | Ⅰ | Ⅱ | Ⅲ | Ⅳ |
| Surface color | light yellow | yellow | brownish yellow | yellowish brown |
| Section color | milky | yellow white | brownish yellow | Yellowish brown |
| Odor | burnt aroma | burnt aroma(slight) | rancidity (slight) | rancidity |
| Oily condition | none | mild | moderate | severe |

**Determine the evaluation factor set**

Evaluation factor set (M), *M* = {surface color, section color, odor, oily state}.

**Determine the rating set**

Determine the rating set(*N*), *N* = {n1, n2, n3, n4}. (n1:90, n2: 70, n3: 50, n4: 30).

Tab. 4 Weights of sensory evaluation indexes of raw ASA samples

| Evaluation index | Score of each index (points) | | | | | Weight |
| --- | --- | --- | --- | --- | --- | --- |
|  | Surface color | Section color | Odor | Oily state | Total |  |
| Surface color | 10 | 1 | 6 | 2 | 19 | 0.19 |
| Section color | 9 | 10 | 8 | 2 | 29 | 0.29 |
| Odor | 4 | 2 | 10 | 3 | 19 | 0.19 |
| Oily state | 8 | 8 | 7 | 10 | 33 | 0.33 |

*X* _raw_= {0.19,0.29,0.19,0.33}.

Tab. 5 Weights of sensory evaluation indexes of peeled ASA samples

| Evaluation index | Score of each index (points) | | | | | Weight |
| --- | --- | --- | --- | --- | --- | --- |
|  | Surface color | Section color | Odor | Oily state | Total |  |
| Surface color | 10 | 3 | 4 | 4 | 21 | 0.21 |
| Section color | 7 | 10 | 5 | 2 | 24 | 0.24 |
| Odor | 6 | 5 | 10 | 5 | 26 | 0.26 |
| Oily state | 6 | 8 | 5 | 10 | 29 | 0.29 |

*X* _peeled_ = {0.21,0.24,0.26,0.29}.

Tab. 6 Weights of sensory evaluation indexes of fried ASA samples

| Evaluation index | Score of each index (points) | | | | | Weight |
| --- | --- | --- | --- | --- | --- | --- |
|  | Surface  color | Section color | Odor | Oily state | Total |  |
| Surface  color | 10 | 4 | 7 | 3 | 24 | 0.24 |
| Section color | 6 | 10 | 6 | 2 | 24 | 0.24 |
| Odor | 3 | 4 | 10 | 3 | 20 | 0.20 |
| Oily state | 7 | 8 | 7 | 10 | 32 | 0.32 |

*X* _fried_ ={0.24，0.24，0.20，0.32}.

**Evaluation results**

Tab. 7 Sensory evaluation results of ASA samples

| Sample number | Number of people who choose grade of surface color (person) | | | | Number of people who choose grade of section color (person) | | | | Number of people who choose grade of odor (person) | | | | Number of people who choose grade of oily state (person) | | | |
| --- | --- | --- | --- | --- | --- | --- | --- | --- | --- | --- | --- | --- | --- | --- | --- | --- |
|  | n1 | n2 | n3 | n4 | n1 | n2 | n3 | n4 | n1 | n2 | n3 | n4 | n1 | n2 | n3 | n4 |
| S1 | 1 | 8 | 1 | 0 | 9 | 1 | 0 | 0 | 3 | 6 | 1 | 0 | 10 | 0 | 0 | 0 |
| S2 | 5 | 5 | 0 | 0 | 6 | 3 | 1 | 0 | 3 | 7 | 0 | 0 | 8 | 2 | 0 | 0 |
| S3 | 6 | 4 | 0 | 0 | 7 | 3 | 0 | 0 | 5 | 5 | 0 | 0 | 8 | 2 | 0 | 0 |
| S4 | 8 | 2 | 0 | 0 | 8 | 2 | 0 | 0 | 4 | 6 | 0 | 0 | 9 | 1 | 0 | 0 |
| S5 | 8 | 1 | 1 | 0 | 8 | 2 | 0 | 0 | 4 | 6 | 0 | 0 | 10 | 0 | 0 | 0 |
| S6 | 0 | 6 | 2 | 2 | 10 | 0 | 0 | 0 | 3 | 5 | 2 | 0 | 10 | 0 | 0 | 0 |
| S7 | 0 | 5 | 4 | 1 | 10 | 0 | 0 | 0 | 3 | 7 | 0 | 0 | 10 | 0 | 0 | 0 |
| S8 | 7 | 3 | 0 | 0 | 10 | 0 | 0 | 0 | 3 | 7 | 0 | 0 | 10 | 0 | 0 | 0 |
| S9 | 1 | 7 | 2 | 0 | 9 | 1 | 0 | 0 | 4 | 5 | 1 | 0 | 10 | 0 | 0 | 0 |
| S10 | 2 | 6 | 2 | 0 | 9 | 1 | 0 | 0 | 4 | 6 | 0 | 0 | 10 | 0 | 0 | 0 |
| S11 | 4 | 4 | 2 | 0 | 3 | 7 | 0 | 0 | 1 | 4 | 5 | 0 | 1 | 8 | 1 | 0 |
| S12 | 0 | 7 | 3 | 0 | 4 | 6 | 0 | 0 | 1 | 6 | 2 | 1 | 3 | 7 | 0 | 0 |
| S13 | 5 | 5 | 0 | 0 | 3 | 7 | 0 | 0 | 5 | 2 | 2 | 1 | 1 | 7 | 2 | 0 |
| S14 | 0 | 2 | 7 | 1 | 1 | 9 | 0 | 0 | 4 | 2 | 4 | 0 | 0 | 9 | 1 | 0 |
| S15 | 0 | 4 | 6 | 0 | 0 | 8 | 2 | 0 | 4 | 2 | 3 | 1 | 0 | 3 | 6 | 1 |
| S16 | 0 | 5 | 3 | 2 | 0 | 0 | 8 | 2 | 2 | 0 | 6 | 2 | 0 | 1 | 4 | 5 |
| S17 | 1 | 2 | 4 | 3 | 0 | 1 | 6 | 3 | 5 | 0 | 2 | 3 | 0 | 0 | 4 | 6 |
| S18 | 0 | 1 | 5 | 4 | 0 | 0 | 6 | 4 | 0 | 1 | 4 | 5 | 0 | 0 | 4 | 6 |
| S19 | 0 | 1 | 2 | 7 | 0 | 0 | 1 | 9 | 0 | 1 | 4 | 5 | 0 | 0 | 1 | 9 |
| S20 | 0 | 0 | 4 | 6 | 0 | 0 | 2 | 8 | 0 | 0 | 4 | 6 | 0 | 0 | 1 | 9 |
| S21 | 0 | 0 | 2 | 8 | 0 | 0 | 0 | 10 | 0 | 0 | 1 | 9 | 0 | 0 | 0 | 10 |
| S22 | 0 | 0 | 2 | 8 | 0 | 0 | 2 | 8 | 0 | 0 | 1 | 9 | 0 | 0 | 1 | 9 |
| D1 | 7 | 3 | 0 | 0 | 8 | 2 | 0 | 0 | 6 | 3 | 1 | 0 | 5 | 5 | 0 | 0 |
| D2 | 9 | 1 | 0 | 0 | 7 | 3 | 0 | 0 | 3 | 7 | 0 | 0 | 4 | 5 | 1 | 0 |
| D3 | 9 | 1 | 0 | 0 | 10 | 0 | 0 | 0 | 3 | 6 | 1 | 0 | 3 | 7 | 0 | 0 |
| D4 | 8 | 2 | 0 | 0 | 6 | 4 | 0 | 0 | 4 | 6 | 0 | 0 | 3 | 6 | 1 | 0 |
| D5 | 5 | 5 | 0 | 0 | 7 | 3 | 0 | 0 | 6 | 3 | 1 | 0 | 6 | 4 | 0 | 0 |
| D6 | 10 | 0 | 0 | 0 | 9 | 1 | 0 | 0 | 6 | 4 | 0 | 0 | 6 | 4 | 0 | 0 |
| D7 | 10 | 0 | 0 | 0 | 9 | 1 | 0 | 0 | 3 | 7 | 0 | 0 | 8 | 2 | 0 | 0 |
| D8 | 9 | 1 | 0 | 0 | 9 | 1 | 0 | 0 | 4 | 5 | 1 | 0 | 8 | 2 | 0 | 0 |
| D9 | 10 | 0 | 0 | 0 | 8 | 2 | 0 | 0 | 2 | 6 | 2 | 0 | 5 | 5 | 0 | 0 |
| D10 | 9 | 1 | 0 | 0 | 8 | 2 | 0 | 0 | 4 | 6 | 0 | 0 | 6 | 4 | 0 | 0 |
| D11 | 6 | 4 | 0 | 0 | 6 | 4 | 0 | 0 | 3 | 5 | 2 | 0 | 1 | 8 | 1 | 0 |
| D12 | 4 | 5 | 1 | 0 | 3 | 7 | 0 | 0 | 2 | 5 | 3 | 0 | 2 | 7 | 1 | 0 |
| D13 | 1 | 9 | 0 | 0 | 2 | 8 | 0 | 0 | 3 | 5 | 2 | 0 | 1 | 5 | 4 | 0 |
| D14 | 0 | 9 | 1 | 0 | 2 | 7 | 1 | 0 | 4 | 4 | 2 | 0 | 0 | 5 | 5 | 0 |
| D15 | 0 | 8 | 1 | 1 | 1 | 8 | 1 | 0 | 5 | 3 | 1 | 1 | 0 | 3 | 6 | 1 |
| D16 | 0 | 0 | 9 | 1 | 0 | 1 | 9 | 0 | 4 | 0 | 6 | 0 | 0 | 0 | 3 | 7 |
| D17 | 0 | 1 | 7 | 2 | 0 | 1 | 7 | 2 | 2 | 2 | 4 | 2 | 0 | 0 | 2 | 8 |
| D18 | 0 | 1 | 3 | 6 | 0 | 1 | 2 | 7 | 2 | 0 | 4 | 4 | 0 | 0 | 2 | 8 |
| D19 | 0 | 0 | 4 | 6 | 0 | 1 | 2 | 7 | 0 | 1 | 3 | 6 | 0 | 0 | 1 | 9 |
| D20 | 0 | 0 | 1 | 9 | 0 | 1 | 0 | 9 | 0 | 0 | 3 | 7 | 0 | 0 | 1 | 9 |
| D21 | 0 | 0 | 0 | 10 | 0 | 0 | 1 | 9 | 0 | 0 | 2 | 8 | 0 | 0 | 0 | 10 |
| C1 | 9 | 1 | 0 | 0 | 10 | 0 | 0 | 0 | 7 | 2 | 1 | 0 | 6 | 3 | 1 | 0 |
| C2 | 9 | 1 | 0 | 0 | 10 | 0 | 0 | 0 | 4 | 6 | 0 | 0 | 6 | 4 | 0 | 0 |
| C3 | 10 | 0 | 0 | 0 | 10 | 0 | 0 | 0 | 3 | 7 | 0 | 0 | 7 | 3 | 0 | 0 |
| C4 | 9 | 1 | 0 | 0 | 10 | 0 | 0 | 0 | 2 | 7 | 1 | 0 | 7 | 3 | 0 | 0 |
| C5 | 10 | 0 | 0 | 0 | 8 | 2 | 0 | 0 | 3 | 7 | 0 | 0 | 5 | 5 | 0 | 0 |
| C6 | 6 | 4 | 0 | 0 | 6 | 4 | 0 | 0 | 4 | 5 | 1 | 0 | 2 | 7 | 1 | 0 |
| C7 | 6 | 4 | 0 | 0 | 6 | 4 | 0 | 0 | 4 | 3 | 3 | 0 | 3 | 5 | 2 | 0 |
| C8 | 5 | 5 | 0 | 0 | 2 | 8 | 0 | 0 | 3 | 6 | 1 | 0 | 2 | 6 | 2 | 0 |
| C9 | 6 | 3 | 1 | 0 | 5 | 5 | 0 | 0 | 2 | 6 | 2 | 0 | 4 | 4 | 2 | 0 |
| C10 | 3 | 6 | 1 | 0 | 3 | 6 | 1 | 0 | 3 | 6 | 1 | 0 | 1 | 9 | 0 | 0 |
| C11 | 7 | 3 | 0 | 0 | 4 | 6 | 0 | 0 | 4 | 5 | 1 | 0 | 1 | 6 | 3 | 0 |
| C12 | 3 | 6 | 1 | 0 | 3 | 6 | 1 | 0 | 3 | 3 | 3 | 1 | 1 | 2 | 6 | 1 |
| C13 | 2 | 7 | 1 | 0 | 3 | 5 | 2 | 0 | 2 | 4 | 4 | 0 | 0 | 3 | 7 | 0 |
| C14 | 1 | 9 | 0 | 0 | 1 | 9 | 0 | 0 | 3 | 2 | 5 | 0 | 0 | 5 | 4 | 1 |
| C15 | 1 | 6 | 3 | 0 | 2 | 6 | 2 | 0 | 4 | 3 | 3 | 0 | 1 | 5 | 4 | 0 |
| C16 | 0 | 1 | 7 | 2 | 0 | 1 | 7 | 2 | 4 | 2 | 3 | 1 | 0 | 0 | 5 | 5 |
| C17 | 0 | 1 | 6 | 3 | 0 | 0 | 7 | 3 | 2 | 1 | 6 | 1 | 0 | 0 | 2 | 8 |
| C18 | 0 | 0 | 4 | 6 | 0 | 0 | 4 | 6 | 1 | 1 | 3 | 5 | 0 | 0 | 2 | 8 |
| C19 | 0 | 0 | 2 | 8 | 0 | 0 | 2 | 8 | 0 | 1 | 1 | 8 | 0 | 0 | 1 | 9 |
| C20 | 0 | 0 | 1 | 9 | 0 | 0 | 1 | 9 | 0 | 1 | 2 | 7 | 0 | 0 | 0 | 10 |
| C21 | 0 | 0 | 2 | 8 | 0 | 0 | 1 | 9 | 0 | 0 | 3 | 7 | 0 | 0 | 0 | 10 |

**Fuzzy evaluation matrix and calculation of score value**

The evaluator refers to Table 3-1 and the scale to evaluate each index of the sample. After the sensory evaluation, the score of each index is counted. Divide the votes of each level by the total number of assessors (10 people) to get a fuzzy relationship matrix R,

R=$\left[ \begin{matrix} \begin{matrix} r11 & r12 \\ r21 & r22 \end{matrix} & \begin{matrix} r13 & r14 \\ r23 & r24 \end{matrix} \\ \begin{matrix} r31 & r32 \\ r41 & r42 \end{matrix} & \begin{matrix} r33 & r34 \\ r43 & r44 \end{matrix} \end{matrix} \right]$/10（Formula 1）

Among them, ri1, ri2, ri3, and ri4 represented the number of votes obtained by the corresponding level of the i-th index.

According to the fuzzy transformation principle, the comprehensive evaluation results of bitter almonds were as follows:

Y = X·R = X·$\left[ \begin{matrix} \begin{matrix} r11 & r12 \\ r21 & r22 \end{matrix} & \begin{matrix} r13 & r14 \\ r23 & r24 \end{matrix} \\ \begin{matrix} r31 & r32 \\ r41 & r42 \end{matrix} & \begin{matrix} r33 & r34 \\ r43 & r44 \end{matrix} \end{matrix} \right]$/10（Formula 2）

Multiply each quantity of the fuzzy comprehensive evaluation result by its corresponding score, and add them to get the total score of each sample.

T=Y*K=Y*$\left[ \begin{matrix} 90 \\ 70 \\ 50 \\ 30 \end{matrix} \right]$（Formula3）

Tab. 8 Sensory evaluation score of ASA samples

| Sample number | Score | Sample number | Score | Sample number | Score |
| --- | --- | --- | --- | --- | --- |
| S1 | 82.58 | D1 | 82.28 | C1 | 84.72 |
| S2 | 81.22 | D2 | 80.44 | C2 | 84.56 |
| S3 | 83.52 | D3 | 81.36 | C3 | 85.28 |
| S4 | 85.14 | D4 | 79.48 | C4 | 84.00 |
| S5 | 85.42 | D5 | 81.54 | C5 | 83.04 |
| S6 | 80.50 | D6 | 85.12 | C6 | 77.60 |
| S7 | 81.26 | D7 | 84.72 | C7 | 76.80 |
| S8 | 86.20 | D8 | 84.30 | C8 | 74.16 |
| S9 | 82.58 | D9 | 80.94 | C9 | 76.08 |
| S10 | 83.34 | D10 | 83.18 | C10 | 73.36 |
| S11 | 70.98 | D11 | 75.92 | C11 | 75.20 |
| S12 | 72.02 | D12 | 72.76 | C12 | 66.64 |
| S13 | 73.36 | D13 | 70.16 | C13 | 65.68 |
| S14 | 66.50 | D14 | 68.20 | C14 | 66.32 |
| S15 | 60.90 | D15 | 65.14 | C15 | 67.52 |
| S16 | 48.10 | D16 | 50.16 | C16 | 49.44 |
| S17 | 47.92 | D17 | 46.54 | C17 | 44.08 |
| S18 | 41.06 | D18 | 40.38 | C18 | 38.32 |
| S19 | 35.04 | D19 | 36.78 | C19 | 33.76 |
| S20 | 34.86 | D20 | 33.52 | C20 | 32.56 |
| S21 | 31.14 | D21 | 31.52 | C21 | 32.64 |
| S22 | 32.96 |  |  |  |  |

Tab. 9 Sensory evaluation score for different rancid levels of ASA samples

| Degree | Sensory evaluation score |
| --- | --- |
| Ⅰ | ＞80 |
| Ⅱ | 60~80 |
| Ⅲ | 40~60 |
| Ⅳ | ＜40 |

Tab. 10 Classification of different rancid levels of ASA samples

| Degree | Sample number |
| --- | --- |
| Ⅰ | S1-S10；D1-D10；C1-C5； |
| Ⅱ | S11-S15；D11-D15；C6-C15 |
| Ⅲ | S16-S17；D16-D17；C16-C17 |
| Ⅳ | S18-S22；D18-D21；C18-C21 |

**Part Three: Color Determination of ASA Samples**

Tab. 11 Color measurement results of ASA samples

| Sample number | Surface color | | | Section color | | |
| --- | --- | --- | --- | --- | --- | --- |
|  | L* | a* | b* | L* | a* | b* |
| S1 | 82.8609 | 1.6112 | 18.1403 | 86.4185 | 0.1930 | 16.1970 |
| S2 | 81.6904 | 1.8487 | 17.0928 | 85.7560 | 0.2580 | 16.5970 |
| S3 | 81.7148 | 3.0591 | 18.7418 | 87.4190 | 0.2955 | 16.3190 |
| S4 | 79.3510 | 1.4914 | 17.1116 | 86.1121 | 0.7579 | 16.9121 |
| S5 | 82.8138 | 2.1448 | 17.9163 | 85.1076 | 0.9063 | 17.8056 |
| S6 | 84.7600 | 1.5359 | 17.1912 | 87.9647 | -0.7206 | 17.1388 |
| S7 | 84.0994 | 1.6240 | 17.7149 | 87.9275 | -0.3630 | 17.1510 |
| S8 | 84.5152 | 1.3623 | 17.1426 | 87.2355 | -0.1460 | 17.7010 |
| S9 | 82.4268 | 1.5539 | 16.9325 | 87.3005 | -0.3175 | 18.3830 |
| S10 | 83.5065 | 1.9664 | 17.3957 | 87.0505 | 0.0397 | 18.2930 |
| S11 | 79.9048 | 1.9315 | 19.6716 | 82.9056 | 0.5317 | 20.7117 |
| S12 | 78.5555 | 2.9175 | 20.9288 | 81.1236 | 1.0645 | 21.8273 |
| S13 | 78.1438 | 2.7910 | 20.6164 | 79.4300 | 1.2715 | 23.0800 |
| S14 | 78.3210 | 3.6865 | 23.2282 | 76.5664 | 2.7936 | 25.9871 |
| S15 | 70.0070 | 5.8925 | 25.3265 | 74.2733 | 4.1160 | 27.0340 |
| S16 | 67.6635 | 7.1482 | 25.4404 | 47.0110 | 7.2480 | 22.4190 |
| S17 | 64.6301 | 8.2107 | 27.2283 | 42.1330 | 7.3990 | 18.7590 |
| S18 | 58.2845 | 10.6687 | 29.8396 | 43.9480 | 7.2370 | 19.1890 |
| S19 | 55.0498 | 11.3892 | 28.5442 | 34.2783 | 7.9525 | 11.0305 |
| S20 | 47.2147 | 13.2431 | 27.6808 | 32.0085 | 7.5183 | 12.3967 |
| S21 | 42.0125 | 13.5985 | 24.2619 | 29.9330 | 8.1510 | 10.4410 |
| S22 | 43.3463 | 13.4033 | 25.1123 | 29.8760 | 11.5830 | 13.8740 |
| D1 | 82.2969 | 1.4340 | 15.8108 | 86.3446 | -0.2462 | 17.8008 |
| D2 | 80.1591 | 1.6396 | 15.4120 | 84.5280 | 0.2920 | 21.3873 |
| D3 | 83.0044 | 1.6093 | 14.9533 | 85.4561 | 0.2617 | 20.9761 |
| D4 | 83.3590 | 1.6836 | 15.2737 | 85.6595 | -0.2540 | 19.0845 |
| D5 | 81.8426 | 1.6465 | 15.5955 | 84.3730 | 0.2991 | 20.1020 |
| D6 | 83.3507 | 1.1316 | 17.5195 | 85.0121 | -0.1311 | 23.7942 |
| D7 | 78.5216 | 1.6815 | 18.8189 | 86.0142 | -0.5589 | 22.1984 |
| D8 | 82.7256 | 2.3346 | 20.4012 | 85.0739 | 0.4767 | 21.6328 |
| D9 | 80.7369 | 3.1530 | 21.2467 | 85.1395 | 0.8005 | 23.3410 |
| D10 | 82.4238 | 1.5387 | 19.4192 | 83.3273 | 1.0157 | 23.5005 |
| D11 | 80.1671 | 2.4112 | 17.7931 | 83.1038 | 0.6019 | 21.8538 |
| D12 | 79.3145 | 3.1278 | 19.4700 | 82.3825 | 0.2883 | 22.6033 |
| D13 | 77.1766 | 3.6345 | 20.4838 | 78.1691 | 2.6364 | 27.0100 |
| D14 | 75.2924 | 4.2264 | 22.3421 | 73.9336 | 3.8971 | 28.3007 |
| D15 | 75.1251 | 4.3809 | 22.0801 | 72.3208 | 5.2638 | 30.0015 |
| D16 | 72.4571 | 5.7445 | 24.4930 | 54.2956 | 6.4213 | 25.9900 |
| D17 | 69.6853 | 6.9070 | 26.3346 | 46.5118 | 6.0929 | 20.8865 |
| D18 | 66.8007 | 8.0456 | 27.8024 | 43.5019 | 7.4619 | 20.0931 |
| D19 | 64.4821 | 8.1389 | 26.8744 | 42.8089 | 8.2011 | 19.1532 |
| D20 | 62.5534 | 9.4982 | 28.2789 | 42.0692 | 7.4854 | 17.7046 |
| D21 | 58.2230 | 10.4720 | 27.2815 | 37.5622 | 8.2322 | 14.8828 |
| D22 | 58.6894 | 7.4454 | 22.7619 | 48.5225 | 10.6083 | 23.0458 |
| C1 | 76.4251 | 2.3965 | 16.0040 | 86.5647 | -0.5671 | 16.8571 |
| C2 | 76.0091 | 2.2769 | 15.9685 | 85.7220 | -0.2350 | 17.7715 |
| C3 | 80.5098 | 2.1023 | 15.3600 | 85.8680 | -0.1495 | 19.5195 |
| C4 | 81.6097 | 1.9768 | 15.1484 | 86.3370 | -0.3080 | 18.5765 |
| C5 | 79.4202 | 2.7021 | 17.2825 | 85.0500 | 0.2368 | 18.9612 |
| C6 | 79.8331 | 2.9735 | 20.8605 | 81.9694 | 1.1661 | 25.8661 |
| C7 | 76.4303 | 3.5400 | 22.3718 | 84.5450 | -0.0750 | 23.9894 |
| C8 | 78.3402 | 3.4348 | 22.1992 | 84.7742 | 1.0192 | 24.6358 |
| C9 | 78.9814 | 3.2328 | 21.1653 | 85.5089 | 0.6906 | 23.0856 |
| C10 | 78.3504 | 3.6719 | 21.3616 | 82.3885 | 1.4307 | 24.5004 |
| C11 | 77.8623 | 3.3487 | 18.2252 | 74.3669 | -0.5323 | 20.2154 |
| C12 | 74.5664 | 3.6789 | 17.9135 | 80.0438 | 0.2900 | 21.8115 |
| C13 | 72.4057 | 4.1207 | 20.1998 | 78.3885 | 0.5662 | 22.4038 |
| C14 | 71.4725 | 4.5247 | 21.3894 | 79.2394 | 1.2428 | 24.1711 |
| C15 | 70.5243 | 5.1898 | 22.7575 | 77.0967 | 3.6278 | 26.2450 |
| C16 | 69.7300 | 5.6602 | 23.8385 | 52.7150 | 4.0325 | 21.2738 |
| C17 | 64.9637 | 7.4218 | 25.8718 | 48.3023 | 3.2385 | 19.1562 |
| C18 | 63.7969 | 8.3154 | 27.6341 | 48.1306 | 5.0244 | 19.8638 |
| C19 | 62.4632 | 9.1953 | 27.4979 | 44.0850 | 6.0172 | 18.8456 |
| C20 | 59.6325 | 10.1386 | 27.9317 | 39.6024 | 7.0271 | 15.8365 |
| C21 | 57.9540 | 10.5309 | 27.7370 | 38.0739 | 7.8606 | 15.4911 |
| C22 | 52.2663 | 6.5960 | 20.1576 | 49.8810 | 10.4930 | 23.3980 |

Tab. 12 Machine learning recognition results of ASA samples with different rancid levels based on powder color

| No. | Machine learning | Rate of positive judgment（%） | |
| --- | --- | --- | --- |
|  |  | Ten-fold crossover validation | External test set validation |
| 1 | Naive Bayes | 89.86* | 85.71* |
| 2 | SVM | 76.81 | 78.57 |
| 3 | Logistic | 84.06 | 64.29 |
| 4 | Multiple Layer Perception | 86.96 | 57.14 |
| 5 | RBFNetwork | 85.51 | 71.43 |
| 6 | IBK | 85.51 | 57.14 |
| 7 | KStar | 85.51 | 64.29 |
| 8 | FilteredClassifier | 85.51 | 57.14 |
| 9 | Randomcommittee | 84.06 | 71.43 |
| 10 | J48 | 79.71 | 64.29 |
| 11 | LMT | 85.51 | 64.29 |
| 12 | Random Forest | 86.96 | 71.43 |

* the highest rate of positive judgment within 12 machine learning algorithms.

Tab. 13 Machine learning recognition results of ASA samples with different rancid levels based on section color

| No. | Machine learning | Rate of positive judgment（%） | |
| --- | --- | --- | --- |
|  |  | Ten-fold crossover validation | External test set validation |
| 1 | Naive Bayes | 85.51 | 64.29 |
| 2 | SVM | 76.81 | 64.29 |
| 3 | Logistic | 82.61 | 78.57* |
| 4 | Multiple Layer Perception | 85.51 | 78.57* |
| 5 | RBFNetwork | 78.26 | 78.57* |
| 6 | IBK | 84.06 | 64.29 |
| 7 | KStar | 81.16 | 71.43 |
| 8 | FilteredClassifier | 82.61 | 71.43 |
| 9 | Randomcommittee | 82.61 | 71.43 |
| 10 | J48 | 81.16 | 71.43 |
| 11 | LMT | 88.41 | 64.29 |
| 12 | Random Forest | 86.96* | 71.43 |

* the highest rate of positive judgment within 12 machine learning algorithms.

**Part Four: Odor Fingerprint of ASA Samples**

Tab. 14 Identification results of samples with different rancid levels

| No. | Machine learning | Rate of positive judgment（%） | |
| --- | --- | --- | --- |
|  |  | Ten-fold crossover validation | External test set validation |
| 1 | Naive Bayes | 82.05 | 62.50 |
| 2 | SVM | 66.67 | 37.50 |
| 3 | Logistic | 97.44* | 87.50* |
| 4 | Multiple Layer Perception | 94.87 | 75.00 |
| 5 | RBFNetwork | 89.74 | 62.50 |
| 6 | IBK | 92.31 | 50.00 |
| 7 | KStar | 94.87 | 50.00 |
| 8 | FilteredClassifier | 92.31 | 62.50 |
| 9 | RandomCommittee | 89.74 | 50.00 |
| 10 | J48 | 92.31 | 50.00 |
| 11 | LMT | 89.74 | 75.00 |
| 12 | Random Forest | 87.18 | 50.00 |

* the highest rate of positive judgment within 12 machine learning algorithms.

**Part Five: Determination of Amygdalin Content in ASA Samples**

Fig. 1 Standard curve of amygdalin in ASA samples

Tab. 15 Content of amygdalin in 22 batches of ASA samples

| Sample number | Content（%） | Sample number | Content（%） | Sample number | Content（%） |
| --- | --- | --- | --- | --- | --- |
| S1 | 5.07 | D1 | 3.56 | C1 | 3.16 |
| S2 | 5.03 | D2 | 3.53 | C2 | 3.41 |
| S3 | 4.76 | D3 | 3.10 | C3 | 3.38 |
| S4 | 3.85 | D4 | 3.26 | C4 | 3.20 |
| S5 | 3.73 | D5 | 2.28 | C5 | 2.27 |
| S6 | 4.83 | D6 | 2.31 | C6 | 2.13 |
| S7 | 4.82 | D7 | 2.61 | C7 | 2.07 |
| S8 | 4.87 | D8 | 1.45 | C8 | 2.37 |
| S9 | 3.95 | D9 | 1.22 | C9 | 1.89 |
| S10 | 4.14 | D10 | 2.07 | C10 | 2.32 |
| S11 | 5.09 | D11 | 3.22 | C11 | 3.23 |
| S12 | 4.38 | D12 | 2.86 | C12 | 2.62 |
| S13 | 3.29 | D13 | 2.66 | C13 | 2.20 |
| S14 | 2.24 | D14 | 2.44 | C14 | 2.23 |
| S15 | 1.53 | D15 | 2.51 | C15 | 2.03 |
| S16 | 0.58 | D16 | 1.74 | C16 | 1.25 |
| S17 | 0.13 | D17 | 0.95 | C17 | 0.57 |
| S18 | 0.02 | D18 | 0.73 | C18 | 0.32 |
| S19 | 0.02 | D19 | 0.31 | C19 | 0.17 |
| S20 | 0.00 | D20 | 0.25 | C20 | 0.25 |
| S21 | 0.00 | D21 | 0.12 | C21 | 0.07 |
| S22 | 0.00 | D22 | 0.06 | C22 | 0.06 |

Tab. 16 Content of Amygdalin in ASA samples with different rancid degree

| Rancid degree | Content of amygdalin | | |
| --- | --- | --- | --- |
|  | Raw sample (%) | Peeled sample (%) | Fried sample (%) |
| Ⅰ | 3.73~5.07 | 1.22~3.56 | 2.27~3.41 |
| Ⅱ | 1.53~5.09 | 2.44~3.22 | 1.89~3.23 |
| Ⅲ | 0.13~0.58 | 0.95~1.74 | 0.57~1.25 |
| Ⅳ | 0~0.02 | 0.06~0.73 | 0.06~0.32 |

**Part Six: Determination of Peroxide Value of ASA Samples**

Tab. 17 Peroxide value in 22 batches of ASA samples

| Sample number | Peroxide value | Sample number | Peroxide value | Sample number | Peroxide value |
| --- | --- | --- | --- | --- | --- |
| S1 | 0.0051 | D1 | 0.0079 | C1 | 0.0135 |
| S2 | 0.0020 | D2 | 0.0127 | C2 | 0.0092 |
| S3 | 0.0042 | D3 | 0.0127 | C3 | 0.0099 |
| S4 | 0.0045 | D4 | 0.0085 | C4 | 0.0117 |
| S5 | 0.0045 | D5 | 0.0085 | C5 | 0.0116 |
| S6 | 0.0048 | D6 | 0.0093 | C6 | 0.0054 |
| S7 | 0.0051 | D7 | 0.0106 | C7 | 0.0056 |
| S8 | 0.0120 | D8 | 0.0134 | C8 | 0.0092 |
| S9 | 0.0134 | D9 | 0.0130 | C9 | 0.0085 |
| S10 | 0.0169 | D10 | 0.0155 | C10 | 0.0085 |
| S11 | 0.0131 | D11 | 0.0085 | C11 | 0.0099 |
| S12 | 0.0085 | D12 | 0.0113 | C12 | 0.0134 |
| S13 | 0.0310 | D13 | 0.0063 | C13 | 0.0099 |
| S14 | 0.0109 | D14 | 0.0106 | C14 | 0.0063 |
| S15 | 0.0134 | D15 | 0.0113 | C15 | 0.0113 |
| S16 | 0.0127 | D16 | 0.0123 | C16 | 0.0134 |
| S17 | 0.0127 | D17 | 0.0144 | C17 | 0.0204 |
| S18 | 0.0120 | D18 | 0.0193 | C18 | 0.0254 |
| S19 | 0.0183 | D19 | 0.0154 | C19 | 0.0183 |
| S20 | 0.0226 | D20 | 0.0190 | C20 | 0.0190 |
| S21 | 0.0176 | D21 | 0.0226 | C21 | 0.0212 |
| S22 | 0.0211 | D22 | 0.0247 | C22 | 0.0233 |

Tab. 18 Peroxide value in ASA samples of different rancid degrees

| Rancid degree | Peroxide value | | |
| --- | --- | --- | --- |
|  | Raw sample | Peeled sample | Fried sample |
| Ⅰ | 0.002~ 0.0169 | 0.0078~ 0.0155 | 0.0092~ 0.0135 |
| Ⅱ | 0.0085~ 0.031 | 0.0063~ 0.0113 | 0.0054~ 0.0134 |
| Ⅲ | 0.0127 | 0.0123~ 0.0144 | 0.0134~ 0.0204 |
| Ⅳ | 0.012~ 0.0226 | 0.0154~ 0.0247 | 0.0183~ 0.0254 |

**Part Six: Identification of Volatile Components in ASA Samples**

Tab. 19 Volatile components in odor and their relative percentages of raw ASA samples（%）

| Name of chemical compound | Sample number | | | | | Structural type |
| --- | --- | --- | --- | --- | --- | --- |
|  | S1 | S5 | S16 | | S22 |  |
| Dimethyl ether | 0.32 | - | - | - | | ethers |
| 1,3-Benzodioxole, 4-methoxy-6-(2-propenyl) | - | - | 0.14 | 13.34 | | ethers |
| Benzaldehyde | 66.19 | 79.38 | 94.86 | 36.45 | | aldehydes |
| Nonanal | - | - | - | 1.12 | | aldehydes |
| Heptane, 2,2,4,6,6-pentamethyl | 3.91 | 2.67 | 0.12 | 1.71 | | alkanes |
| Dodecane | 0.62 | 0.57 | 0.04 | 0.80 | | alkanes |
| Tritetracontane | 0.37 | 0.22 | 0.05 | 2.33 | | alkanes |
| Benzyl Alcohol | 17.76 | 10.99 | 3.80 | 6.24 | | alcohols |
| 3-Cyclohexen-1-ol, 4-methyl-1-(1-methylethyl) | - | 0.32 | - | - | | alcohols |
| Phytol | 0.36 | 0.20 | - | - | | alcohols |
| 1-Decanol, 2-hexyl | - | 0.21 | 0.02 | 0.58 | | alcohols |
| Benzyl glycolate | - | - | - | 0.72 | | esters |
| Acetic acid, phenylmethyl ester | 1.57 | 1.28 | 0.06 | - | | esters |
| 1,2-Benzenedicarboxylic acid, mono(2-ethylhexyl) ester | 4.47 | 1.43 | 0.11 | - | | esters |
| Didodecyl phthalate | - | - | - | 0.45 | | esters |
| Terephthalic acid, di(2-ethylhexyl) ester | 3.55 | 1.35 | 0.10 | 0.48 | | esters |
| 3-Carene | - | 0.27 | - | - | | alkene |
| 3,5-Dimethyladamantan-1-ylamine | - | - | - | 1.28 | | amines |
| N-Methoxy-N-methylbenzamide | 0.88 | 0.86 | 0.31 | 18.35 | | amines |
| Mandelamide | - | - | 0.15 | 11.21 | | amines |
| 1-Propanone, 2-bromo-1-phenyl | - | - | 0.09 | 1.06 | | ketones |
| 3-Phenyl-1-indanone | - | - | - | 0.67 | | ketones |
| 3,4-Dimethoxytoluene | - | - | - | 0.93 | | aromatic |
| Benzene, 1,2-(methylenedioxy)-4-propenyl-, (E) | - | - | 0.07 | 0.66 | | aromatic |
| 1-Octadecanesulphonyl chloride | - | - | 0.04 | 1.63 | | other |
| cis-13-Octadecenoic acid | - | 0.27 | 0.03 | - | | other |

Tab. 20 Volatile components in odor and their relative percentages of peeled ASA samples（%）

| Name of chemical compound | | Sample number | | | | | | Structural type |
| --- | --- | --- | --- | --- | --- | --- | --- | --- |
|  |  | D1 | D5 | | D16 | D21 | D22 |  |
| Benzaldehyde | | 91.94 | | 79.63 | 90.36 | 70.91 | 5.99 | aldehyde |
| Nonanal | | - | | - | 0.07 | 0.69 | 0.87 | aldehyde |
| Heptane, 2,2,4,6,6-pentamethyl | | - | | 0.71 | 0.13 | 1.79 | 0.98 | alkanes |
| Dodecane | | - | | - | 0.03 | 0.24 | - | alkanes |
| Tritetracontane | | - | | 0.15 | 0.63 | 1.71 | 15.95 | alkanes |
| Eicosane | | - | | - | - | 2.79 | - | alkanes |
| Tetratetracontane | | - | | - | - | 3.82 | - | alkanes |
| Cyclohexene, 1-methyl-4-(1-methylethylidene) | | - | | 0.26 | - | - | - | alkenes |
| 3-Carene | | - | | 0.68 | - | - | - | alkenes |
| Copaene | | - | | 0.16 | 0.04 | 0.24 | - | alkenes |
| 1-Hexacosene | | - | | - | - | - | 0.47 | alkenes |
| 17-Pentatriacontene | | - | | - | 0.29 | 0.88 | 12.48 | alkenes |
| Benzene, 1-methyl-2-(1-methylethyl) | | - | | 0.27 | - | - | - | aromatics |
| 3,4-Dimethoxytoluene | | 0.15 | | 0.32 | 0.10 | 0.27 | - | aromatics |
| Benzene, 1,2-(methylenedioxy)-4-propenyl-, (E) | - | | | 0.38 | 0.03 | 0.22 | 2.43 | aromatics |
| 1-Cyclohexene-1-methanol, 4-(1-methylethenyl) | | - | | - | - | - | - | alcohols |
| Benzyl Alcohol | | 3.67 | | 14.85 | 5.40 | 2.60 | 3.15 | alcohols |
| 3-Cyclohexen-1-ol, 4-methyl-1-(1-methylethyl) | | - | | 0.64 | 0.03 | 0.22 | - | alcohols |
| 1-Decanol, 2-hexyl | | - | | - | 0.36 | 1.54 | 4.24 | alcohols |
| Phytol | | - | | - | 0.05 | 0.29 | 0.54 | alcohols |
| 1-Hentetracontanol | | - | | - | - | - | 5.88 | alcohols |
| Dodecyl acrylate | | - | | - | 0.02 | - | - | esters |
| Acetic acid, phenylmethyl ester | | - | | - | 0.08 | - | - | esters |
| 1,2-Benzenedicarboxylic acid, mono(2-ethylhexyl) ester | | 0.44 | | 0.49 | - | 0.21 | 8.82 | esters |
| Didodecyl phthalate | | - | | - | - | 1.84 | - | esters |
| Terephthalic acid, di(2-ethylhexyl) ester | | 0.43 | | 0.32 | - | - | 9.28 | esters |
| 3,5-Dimethyladamantan-1-ylamine | | 3.36 | | - | - | - | - | amine |
| N-Methoxy-N-methylbenzamide | | - | | 0.64 | 0.49 | 2.57 | 5.99 | amine |
| 1-Propanone, 2-bromo-1-phenyl | | - | | - | 0.09 | - | - | ketones |
| 1,3-Benzodioxole, 4-methoxy-6-(2-propenyl) | | - | | 0.36 | 0.94 | 2.48 | 12.17 | ethers |
| 1-Octadecanesulphonyl chloride | | - | | 0.16 | 0.85 | 1.72 | 7.67 | other |
| Morpholine,4-octadecyl | | - | | - | - | - | - | other |
| Dothiepin | | - | | - | - | 0.40 | - | other |
| cis-13-Octadecenoic acid | | - | | - | - | 2.58 | 3.07 | other |

Tab. 21 Volatile components in odor and their relative percentages of fried ASA samples（%）

| Name of chemical compound | Sample number | | | | | Structural type |
| --- | --- | --- | --- | --- | --- | --- |
|  | C1 | C5 | C16 | C21 | C22 |  |
| Benzaldehyde | 91.56 | 75.83 | 88.64 | 57.12 | 12.66 | aldehydes |
| Nonanal | 0.12 | 0.27 | 0.85 | 1.14 | 0.76 | aldehydes |
| Heptane, 2,2,4,6,6-pentamethyl | 0.22 | 1.72 | 0.17 | 0.56 | 2.22 | alkanes |
| Dodecane | 0.06 | 0.17 | 0.04 | 0.27 | 0.64 | alkanes |
| Tritetracontane | 0.06 | 0.18 | 0.87 | 5.06 | 12.75 | alkanes |
| Eicosane | - | - | - | - | - | alkanes |
| Benzene, 1-methyl-2-(1-methylethyl) | - | 0.45 | - | - | - | aromatic |
| 3,4-Dimethoxytoluene | 0.23 | 0.26 | 0.10 | 0.35 | 0.49 | aromatic |
| Benzene, 1,2-(methylenedioxy)-4-propenyl-, (E) | 0.20 | 0.75 | 0.25 | 0.30 | 0.73 | aromatic |
| 1-Cyclohexene-1-methanol, 4-(1-methylethenyl) | - | 0.69 | - | 0.41 | - | alcohols |
| Benzyl Alcohol | 6.64 | 13.19 | 6.07 | 5.82 | 7.50 | alcohols |
| 3-Cyclohexen-1-ol, 4-methyl-1-(1-methylethyl) | - | 1.00 | - | 0.28 | 0.51 | alcohols |
| 1-Decanol, 2-hexyl | - | - | 0.38 | 3.88 | 10.22 | alcohols |
| Phytol | - | - | 0.11 | 0.66 | 1.30 | alcohols |
| 1-Hentetracontanol | - | - | 0.12 | 2.35 | 2.88 | alcohols |
| 3-Carene | - | 1.00 | - | - | - | olefins |
| Copaene | - | 0.23 | 0.05 | 0.37 | 0.72 | olefins |
| 1-Hexacosene | - | - | 0.05 | 0.32 | 0.91 | olefins |
| 17-Pentatriacontene | - | - | 0.39 | 4.13 | 7.17 | olefins |
| Dodecyl acrylate | - | - | - | 0.09 | - | esters |
| Acetic acid, phenylmethyl ester | - | 0.08 | 0.07 | - | - | esters |
| 1,2-Benzenedicarboxylic acid, mono(2-ethylhexyl) ester | 0.20 | 0.66 | - | 0.24 | - | esters |
| Didodecyl phthalate | - | - | - | - | - | esters |
| Terephthalic acid, di(2-ethylhexyl) ester | 0.18 | 0.68 | - | 0.20 | - | esters |
| 1-Propanone, 2-bromo-1-phenyl | 0.29 | 0.20 | 0.50 | 0.39 | - | ketones |
| N-Methoxy-N-methylbenzamide | 0.12 | 1.03 | 0.64 | 4.95 | 10.04 | amines |
| 1,3-Benzodioxole, 4-methoxy-6-(2-propenyl) | - | 0.91 | 0.78 | 4.98 | 15.59 | ethers |
| Benzeneacetic acid, alpha-hydroxy-, (S) | 0.06 | - | - | - | - | other |
| 1-Octadecanesulphonyl chloride | - | 0.20 | 1.01 | 6.14 | 12.91 | other |
| Morpholine,4-octadecyl | 0.06 | 0.22 | - | - | - | other |
| Dothiepin | - | - | 0.07 | - | - | other |
